# Identification of Rv1133c (MetE) as a marker of *Mycobacterium tuberculosis* replication and as a highly immunogenic antigen with potential immunodiagnostic power

**DOI:** 10.3389/fimmu.2024.1464923

**Published:** 2024-10-04

**Authors:** Angelo Iacobino, Raffaela Teloni, Carmine Mancone, Francesco Facchiano, Alessandra Di Giamberardino, Cinzia Senatore, Antonio Di Virgilio, Alessio Lanni, Federico Giannoni, Roberto Nisini, Sabrina Mariotti

**Affiliations:** ^1^ Dipartimento di Malattie Infettive, Istituto Superiore di Sanità, Roma, Italy; ^2^ Dipartimento di Medicina Molecolare, Sapienza Università di Roma, Roma, Italy; ^3^ Dipartimento Oncologia e Medicina Molecolare, Istituto Superiore di Sanità, Roma, Italy; ^4^ Centro per la Sperimentazione ed il Benessere Animale, Istituto Superiore di Sanità, Roma, Italy

**Keywords:** tuberculosis, latent infection, vitamin B12, monoclonal antibodies, MetE, Rv1133c, diagnosis

## Abstract

The immunization of mice with the sterile culture medium supernatants of *Mycobacterium tuberculosis* (Mtb) H37Rv permitted the production of several monoclonal antibodies (mAbs) specific for secreted and/or released antigens. Two mAbs bound and immunoprecipitated an 80-kDa protein that was identified by mass spectrometry as Rv1133c, the methionine synthase MetE. The protein MetE is ubiquitous among prokaryota and shows a significant sequence homology in many bacteria. We produced both the full-length recombinant MetE and its N-terminal fragment, whose sequence is more conserved among mycobacteria, to select mAbs recognizing an Mtb-specific region of MetE. Finally, we produced and selected eight mAbs that specifically detect the MetE protein in the supernatant and cell lysate of Mtb and BCG, but not other bacteria such as non-tuberculous mycobacteria (NTM), *Streptococcus pneumoniae, Staphylococcus aureus, Acinetobacter baumanii*, or *Escherichia coli*. Taking advantage of our mAbs, we studied (i) the vitamin B12 dependence for the synthesis of MetE in Mtb and NTM and (ii) the kinetics of MetE production and secretion in supernatants during the *in vitro* reproduced replicative, dormant, and resuscitation cycle of Mtb. Our data demonstrate that dormant Mtb, which are assumed to be prevalent in latent infections, as well as NTM do not produce and secrete MetE. Results indicate an unexpected specificity for Mtb of our anti-MetE mAbs and encourage the use of rMetE and our mAbs as tools for the immunodiagnosis of TB and its stages.

## 1 Introduction

The intracellular bacterium *Mycobacterium tuberculosis* (Mtb) causes tuberculosis (TB), one of the most threatening infectious diseases. The World Health Organization (WHO) estimates that 10.6 million people were infected with Mtb and 1.3 million died of TB in 2022 (https://www.who.int/teams/global-tuberculosis-programme/tb-reports). The immune response to Mtb in TB patients (1) includes both the cellular and humoral arms of the adaptive immune system. The cellular response involves the expansion of CD4 and CD8 T lymphocytes that, upon activation by Mtb specific antigens, contributes to limit Mtb spread by killing infected cells. The humoral response is characterized by the production of antibodies specific for surface-exposed or released Mtb antigens, including cell wall-associated (glyco-lipo)-protein, membrane, extracellular, and protein or carbohydrate antigens of Mtb released in the course of the disease. Antibodies raised against Mtb antigens during TB potentially limit the dissemination of bacilli and may play an important role in the prevention of infection. For example, antibodies may confer protection against mycobacteria by modulating immunity via Fc receptor-mediated phagocytosis (2). In fact, in infections caused by intracellular bacteria such as Mtb, antibodies that bind surface antigens can trigger opsono-phagocytosis during the extracellular phase of the pathogen life cycles (3, 4) or neutralize ligands of cellular receptors. For example, antibodies could interfere with the functions of surface-exposed molecules important for Mtb interactions with its target cells to reach the right intracellular compartment where they can survive in a low acidic and non-proteolytic microenvironment. Besides their protective role, cellular and, to a lesser extent, humoral specific immune responses have been exploited to increase the power of current protocols for the diagnosis of TB (5).

In this work, we immunized mice with the sterile culture medium supernatants (SCMS) of Mtb H37Rv to produce monoclonal antibodies (mAbs) aiming to identify previously undervalued immunogenic antigens. A proteomic analysis of SCMS revealed the most represented Mtb released proteins and the obtained mAbs permitted the identification of the most immunogenic ones.

Here, we focused on one of the proteins recognized with the applied strategy (**Supplementary Figure S1**): Rv1133c (MetE), which was highly represented in the SCMS and induced a robust humoral immune response in mice, allowing the isolation of several MetE-specific mAbs, suggesting its high immunogenicity.

MetE is a cobalamin-independent methionine synthase involved in the final step of methionine biosynthesis: it catalyzes the transfer of a methyl group from 5-methyltetrahydrofolate to homocysteine, resulting in methionine formation (6). This protein has been previously identified in the culture filtrate, membrane protein fraction, and whole cell lysates of *M. tuberculosis* H37Rv (7).

Methionine is important in central metabolism, being involved in many biosynthetic pathways and in the initiation and elongation of translation, and its derivative S-adenosyl-methionine serves as a universal methyl donor. The transfer of methyl group from 5-methyltetrahydrofolate to homocysteine, resulting in methionine formation, can be obtained in Mtb by two enzymes: a vitamin B12-dependent transmethylase, MetH, or by a vitamin B12-independent transmethylase, MetE. In the absence of exogenously supplied vitamin B12, MetE will be the only transmethylase of methionine biosynthesis, making *metE* an essential gene for methionine biosynthesis (8).

Since the MetE protein is ubiquitous among bacteria, as it has a pivotal part in the methionine metabolic pathway, to select mAbs recognizing the specific region of Mtb MetE, we produced both the full-length recombinant MetE and its N-terminal fragment, whose sequence is more conserved among mycobacteria. We showed that the selected mAbs were able to detect MetE protein in the supernatant of Mtb and BCG, but not in the other tested bacteria.

Moreover, we compared the expression of MetE in actively replicating, dormant, and reactivated Mtb, to investigate its role in the different stages of Mtb growth and to hypothesize the possibility that MetE detection in biofluids could help in identifying Mtb infection and in discriminating patients with active TB from individuals with latent TB infection (LTBI).

## 2 Materials and methods

### 2.1 Human samples

Sera from male TB patients and healthy controls (age range, 38–48) were obtained from donors who signed an informed consent to participate in the study. With the exception of one patient who had lymph node localization, the others received a diagnosis of lung TB and were positive in the IGRA test. The study and its protocol were conducted in accordance with the Declaration of Helsinki and approved by the Ethics Committee of Istituto Superiore di Sanità (protocol code PRE-726/16 on 20 August 2016).

### 2.2 Growth of bacteria and SCMS preparation

Aerobic and hypoxic Mtb H37Rv (ATCC 27294) cultures were generated as previously described (9, 10). Briefly, aerobic bacilli were grown at 37°C in agitation at 250 rpm in 7H9 ADC or Dubos Tween-albumin (DTA) broth, while hypoxic bacteria were grown by stirring (120 rpm) in sealed glass tubes containing DTA broth and incubated for up to 25 days at 37°C. Control tubes with 1.5 mg/mL methylene blue as an indicator of oxygen depletion were included. Resuscitated Mtb were analyzed after loosening the cap of hypoxic cultures and left to grow aerobically for 7 more days. Mtb SCMS was obtained by growing Mtb H37Rv aerobically for 25 days, in Sauton minimal medium, at 37°C in agitation at 250 rpm. The culture obtained was centrifugated and the filtered supernatant was used for mice immunization and antibody selection. All the Mtb manipulation was carried out in the BSL-3 laboratory. Other strains used in this work were as follows: *M. bovis BCG* (ATCC 27291), *M. avium* (ATCC 157699), *M. abscessus* (ATCC 19977), and *M. chimaera* (DSM44623), all aerobically grown in 7H9 ADC enriched medium. *S. pneumoniae*, *S. aureus*, and *A. baumanii* were grown in LB medium while *Escherichia coli* BL21 was grown in M9 minimal medium.

### 2.3 Production of recombinant Rv1133c (MetE) proteins in *E. coli*

For the expression in *E. coli* of both MetE full (aa 1–759) protein and N-terminal MetE fragment (aa 1–420), Rv1133c gene was amplified by PCR with the primers MetEFw-MetERv (for rMetE) and MetEFw-MetE_420_Rv (for rMetE_420_) (**Supplementary Table S1**), by using H37Rv genomic DNA as template, prepared by using the GenoLyse protocol (Hain Lifescience GmbH). The obtained amplicons were cloned into the pGEM-T (Promega) intermediate vector and subcloned in the pQE30 expression vector (Qiagen) into BamHI/HindIII restriction sites, as previously described (12). *E. coli* JM109 (Promega) strain was used as expression host. The obtained proteins had an RGS(H)6 tag at the N-terminus and were purified by Ni-NTA affinity chromatography, under native conditions, as described in the QIAexpressionist handbook. The proteins were quantified in densitometry SimplyBlu-stained bands of SDS-PAGE by comparison with known amounts of BSA. The proteins were dialyzed against PBS to remove the elution buffer containing imidazole.

### 2.4 Mice immunization with culture filtrate and recombinant MetE

Six-week-old female pathogen-free BALB/c mice were obtained from Charles River Laboratories (Calco, LC, Italy) and housed in the Istituto Superiore di Sanità. All animal protocols and procedures were performed in accordance with European Union guidelines and Italian legislation (DL26/2014) and have been approved by the Italian Ministry of Healthy and reviewed by the Service for Animal Welfare at ISS (Protocol no. 670/2020-PR of 21 July 2020). Mice were immunized with a subcutaneous injection of 100 µL/mouse of SCMS or of solution containing 50 µg of rMetE mixed with an equal volume of emulsified incomplete Freund’s adjuvant (Millipore-Sigma) at day 0 and at day 14. Serum samples were collected on day 28 and stored at −35°C, as previously described (11, 12). The collected plasma samples were tested by in-house ELISA for the determination of anti-SCMS or anti-MetE specific antibody titers. Mice showing the highest specific antibody levels were injected intravenously with 50 µL/mouse of SCMS or 5 μg/mouse of rMetE without adjuvant at day 40, and after an additional 5 days, mice were sacrificed to isolate the spleen.

### 2.5 Hybridoma fusion, screening, and mAbs purification

The spleens of selected mice were used to isolate SCMS- or MetE-specific mAbs as previously described (11, 12). Briefly, after fusion of spleen cells with the mouse myeloma cell line SP2 (ATCC. Manassas, VA, USA), growing hybridomas were screened for antigen specificity by ELISA using SCMS or rMetE protein-coated plates. The selected polyclonal Ab-producing hybridomas were single-cell-cloned by limiting dilution and the clones producing SCMS or MetE specific mAbs were expanded. The mAbs were purified and concentrated using chromatography cartridges’ protein G columns (Thermo Fisher). The concentration of purified mAbs was evaluated by a NanoPhotometer (Implen) spectrophotometer at 280 nm.

### 2.6 ELISA

The antigen specificity of the immunized mice sera and of the hybridoma supernatants was analyzed by ELISA, as previously described (11, 12). Briefly, 2 μg/mL of rMetE or 5 µg/mL of SCMS proteins were coated overnight at 4°C on 96-well ELISA plates. After washing and blocking with PBS+2% BSA, mouse serum or hybridoma supernatants were incubated for 3 h at 37°C. After washing, alkaline phosphatase (PA)-conjugated goat anti-mouse IgG (Southern Biotech) was added and incubated for 1 h at 37°C. Substrate was added after washing and the developed color was measured as absorbance (ABS) intensity. For the sandwich ELISA, plates were coated overnight with 1 μg/mL of primary mAbs and then incubated with scalar doses of rMetE (from 2,000 to 0.5 ng/mL). A second mAb, from a different subclass, was added at 1 μg/mL and then the sandwich was revealed with an antibody that recognizes the subclass of the second mAb. The most efficient pair (M296/M51) was tested in a sandwich ELISA with a scalar quantity of Mtb SCMS. Results are expressed as optical density value (O.D. at 405 nm) triplicates after subtraction of the blank values. The subclass of isolated mAbs was identified using enzyme-conjugated anti-mouse subclass specific antibody (anti-IgG1, anti-IgG2a, anti-IgG2b, anti-IgG2c, and anti-IgG3; Southern Biotech).

### 2.7 SDS-PAGE and Western blot analysis

SDS-PAGE and WB were carried out to analyze recombinant proteins and SCMS, as previously described (11, 12). Briefly, to detect the MetE protein, isolated mAbs at opportune dilution in TBS containing 0.05% Tween (TBST) were used. Filter-bound immunoglobulins were detected using horseradish peroxidase (HRP)-conjugated goat anti-mouse IgG secondary antibody (Abcam) and signals were detected by Crescendo Western HRP chemiluminescent substrate (Millipore). Culture supernatants of Mtb, BCG, and NTM strains were subjected to SDS-PAGE followed by WB with the specific mAbs at the concentration of 1 μg/mL. Bacterial pellets were lysed by adding SDS-loading buffer containing 50 mM Tris-HCl, pH 6.8, 3% SDS, 50% glycerol, 0.5% bromophenol blue, and 5% β-mercaptoethanol (βME) and then separated by SDS-PAGE.

Human sera were analyzed by WB after SDS-PAGE of rMetE: blotted membranes were incubated with sera diluted 1:100 and detected using HRP-conjugated goat anti-human IgG (Sigma-Aldrich) and signals were detected by Crescendo Western HRP chemiluminescent substrate.

### 2.8 Immunoprecipitation of selected antigen

Selected mAbs were immobilized on chromatography cartridges’ protein G columns (Thermo Fisher) and used to immunoprecipitate the corresponding antigen from Mtb SCMS. Briefly, the culture supernatant of cells producing mAbs was passed through a protein G column, to allow binding of the mAbs to the column. After washing, SCMS from Mtb was also passed through the protein G column with the bound mAbs. After the final washing, the immunocomplex was eluted from the column for the antigen identification by LC-MALDI.

### 2.9 Proteomic and mass spectrometry analysis

Mtb supernatants were prepared as above described, concentrated by ultrafiltration then denatured and electrophoresed by 4%–15% polyacrylamide gel (SDS-PAGE) and stained with Coomassie, according to published protocols with few modifications (Verdoliva et al., 2013). Gel lanes were cut in several pieces, and proteins were reduced, alkylated, and digested by overnight incubation with trypsin (bovine sequencing grade, Roche Applied Science, Monza, IT) according to published protocols (13). The obtained peptide mixtures were consequently analyzed by nano-reversed-phase liquid chromatography tandem mass spectrometry (nRP-LC-MS/MS) using an HPLC Ultimate-3000 (DIONEX, Sunnyvale, CA) connected online with a linear Ion Trap (LTQ, Thermo Electron, San Jose, CA) and then data acquisition and analyses were performed as previously published (13) by setting the data tolerance at 1.5 Da and 1 Da for precursor and fragment ions, respectively. Legitimate identification of peptides through cross-correlation scores (i.e., 1.5 for [M + H]1+, 2.0 for [M + 2H]2+, 2.5 for [M + 3H]3+) and the probability cutoff for randomized identification were set as previously described (14).

### 2.10 Identification of selected antigen by LC-MALDI

Protein immunopurifications were precipitated in ice-cold acetone overnight and resuspended in 40 µL of NH_4_HCO_3_ 50 mM, urea 2 M solution, and digested with 5 µL of trypsin solution (0.2 µg/µL) overnight at 37°C. Peptides were reduced in 10 mM dithiothreitol (DTT) at 56°C for 30 min. Alkylations were performed in 55 mM iodoacetamide (IAA) at room temperature for 20 min in the dark. Peptides were then desalted and filtered through a C18 microcolumn ZipTip and eluted from the C18 bed using 10 μL of 80% acetonitrile/0.1% TFA. The organic component was removed by evaporation in a vacuum centrifuge and peptides were resuspended in 50 µL of 0.1% formic acid and analyzed by LC-MALDI as previously described (15).

### 2.11 qRT-PCR

Mtb was cultured under aerobic and hypoxic conditions, according to Wayne (17), and after bacterial pellet resuspension and disruption, RNA was purified and reverse-transcribed as previously described (19). Briefly, RT-PCR was performed using GoTaq qPCR Master Mix (Promega), and using *16S* as housekeeping gene, relative fold change to aerobic day 3 control was determined using the 2^−^ΔΔCt formula. The primers used to amplify *16S*, *acr*, and *metE* genes are listed in **Supplementary Table S1**.

### 2.12 Flow cytometry

Aerobic Mtb H37Rv cells were incubated with 100 µL of anti-MetE mAbs (1 µg/mL) for 30 min on ice. After three washes, the bacteria were incubated with an anti-mouse IgG FITC-labeled (diluted 1:500) for 30 min on ice. After washes, the cells were acquired by a FACScalibur flow cytometer (Becton Dickinson) equipped with Cellquest Software (Becton Dickinson).

### 2.13 Statistical analysis

All the statistical analyses were performed using the GraphPad Prism software v9 (GraphPad Software) and the *p*-values < 0.05 were considered significant. The half maximal effective concentration (EC_50_) of isolated mAbs was calculated by non-linear regression analysis of log10 of serum dilution plotted versus absorbance.

## 3 Results

### 3.1 Proteomic analysis of Mtb SCMS

Mtb H37Rv was grown for 25 days in Sauton’s minimal medium in the absence of added proteins. Therefore, all the proteins present after the growth could be considered of Mtb origin. SCMS was studied by proteomic analysis that revealed the presence of approximately 180 proteins, 30 of which had a score higher than 100. Among these 30 most represented proteins, 7 had a molecular mass between 75 and 100 kDa, while all the other proteins were smaller (10–70 kDa). The identified proteins with the highest significant scores are reported in **Supplementary Table S2**.

### 3.2 Mice immunization and identification of the Rv1133c/MetE protein

To obtain mAbs specific for released Mtb proteins, we used the strategy summarized in **Supplementary Figure S1**. Mice were immunized twice with SCMS, in the presence of incomplete Freund’s adjuvant. Mice with the higher production of SCMS-specific IgG were selected for mAbs production. Splenocytes were fused with mouse myeloma and hybridomas recognizing (by ELISA and WB) the Mtb SCMS were cloned by limiting dilutions to obtain monoclonal cultures. mAbs were further selected for their capacity to bind the external surface of Mtb cells by flow cytometry (data not shown).

Among the several mAbs obtained, we initially focused on two mAbs, named M35 and M29, isolated from the spleens of two different mice, which recognized a single band of approximately 80 kDa in WB after SDS-PAGE of the SCMS (**Supplementary Figure S2A**). These same mAbs were able to immunoprecipitate a protein with the same molecular mass of approximately 80 kDa from H37Rv cultures (**Supplementary Figure S2B**).

Using the LC-MALDI approach (16), the 80-kDa antigen immunoprecipitated by both mAbs M35 and M29 was identified as the protein MetE (Rv1133c), a vitamin B12-independent methionine synthase, with over 42% sequence coverage (**Supplementary Tables S3A, B**).

Bioinformatic analysis of the amino acid sequence showed that MetE protein is highly conserved among bacteria: sequence alignment revealed 48% amino acid identity with *E. coli* and 79%–86% identity with non-tuberculous mycobacteria (**Supplementary Figures S3A, B**). Interestingly, the N-terminal fragment of Mtb MetE, consisting of aa 1 to aa 420, is notably more different from other bacteria, compared to the C-terminal fragment.

### 3.3 Production of the recombinant full MetE and N-terminal MetE fragment

To gain some insight into the epitopes recognized by our mAbs, we produced the full-length MetE and its N-terminal fragment. To purify the recombinant MetE protein, Rv1133c gene from H37Rv was cloned in pQE30 prokaryotic expression vector (**Supplementary Figure S4A**). Soluble recombinant MetE (rMetE) full protein (aa 1–759) and the N-terminal fragment (aa 1–420, rMetE_420_) were produced by using native conditions. Both recombinant proteins showed the expected molecular mass by SDS-PAGE analysis of 80 kDa (rMetE) and 45 kDa (rMetE_420_), respectively (**Supplementary Figure S4B**). WB performed using the mAb M35 indicated that (1) the recombinant proteins that we obtained are reasonably identical to those secreted by Mtb, since mAb M35 also recognizes and immunoprecipitates the MetE protein present in Mtb culture supernatant, and (2) the epitope recognized by this mAb is located in the more Mtb-specific sequence in the N-terminal fragment of MetE (**Supplementary Figure S4B**).

### 3.4 Additional mAbs raised with rMetE

**Figure 1:**
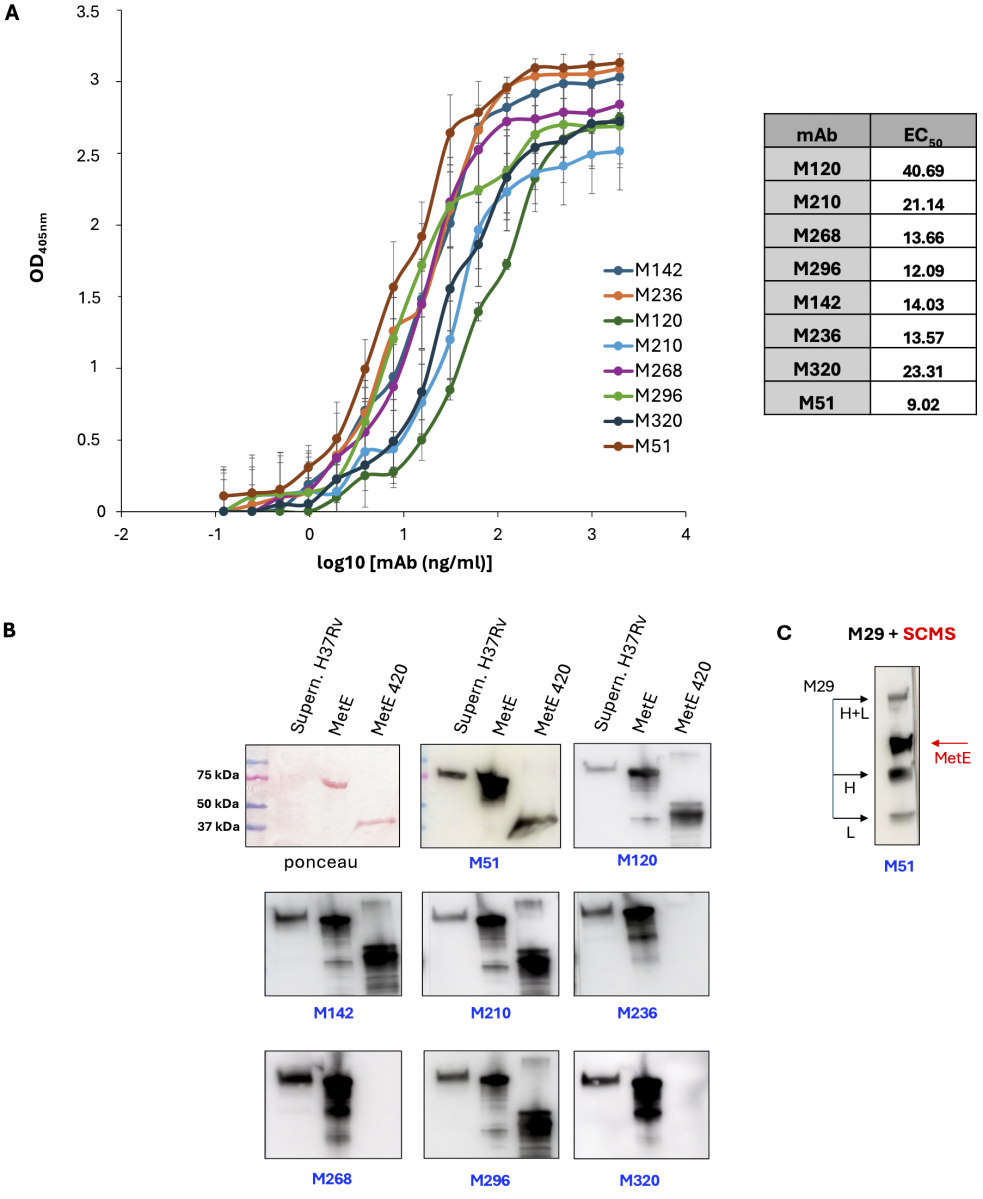
Characterization of MetE-specific monoclonal antibodies. **(A)** mAbs binding affinity to rMetE and EC_50_ (table) measured in ELISA with rMetE-coated plates; error bars indicate standard deviations of technical triplicates from a representative experiment repeated three times. One experiment representative of two experiments is shown. **(B)** SDS-PAGE Ponceau stained culture filtrate H37Rv supernatant, rMetE, and rMetE420 and WB analysis with the different mAbs. One experiment representative of three experiments is shown. **(C)** The immunoprecipitated complex mAb M29-SCMS supernatant of H37Rv was separated by SDS-PAGE followed by WB performed using the mAb M51 as primary antibody. Black arrows indicate the light (L) chain, heavy (H) chain, and L+H chains of mAb M29; red arrow indicates the 80-kDa MetE protein. One experiment representative of three experiments is shown.

The rMetE was used to immunize mice and to isolate eight different additional mAbs that showed high binding affinity to rMetE protein, as measured by EC_50_ and reported in **Figure 1A**. The EC_50_ of all the mAbs showed values ranging from 40 to 9 ng/mL, with the mAb M51 (EC_50_ 9.02 ng/mL) resulting in the mAb with the strongest binding affinity.

All the mAbs, except M236, M268, and M320, were able to recognize both the full-length and the rMetE_420_ fragment, indicating that they bind epitopes localized in the more Mtb-specific N-terminal region of MetE (**Figure 1B**). Finally, all mAbs were IgG1, except for the mAb M296 that belongs to the IgG2b subclass (data not shown).

**Figure 1B** also reports that all the mAbs of the new series recognize the rMetE protein used as antigen for immunization, as well as the Mtb secreted MetE protein present in the culture supernatant of Mtb H37Rv. Moreover, we showed that mAb M51 binds the MetE immunoprecipitated from Mtb culture supernatant by mAb M29 (**Figure 1C**), confirming (i) the good quality and the right conformation of the native recombinant protein that we produced and (ii) that these two high-specific mAbs recognize different, non-overlapping epitopes in the more Mtb-specific sequence of MetE. Moreover, this result indicates that these two mAbs can be used to detect MetE in biofluids. The same result was obtained using the mAbs M51 and M296 (data not shown).

### 3.5 Specificity of mAbs for Mtb and BCG

**Figure 2:**
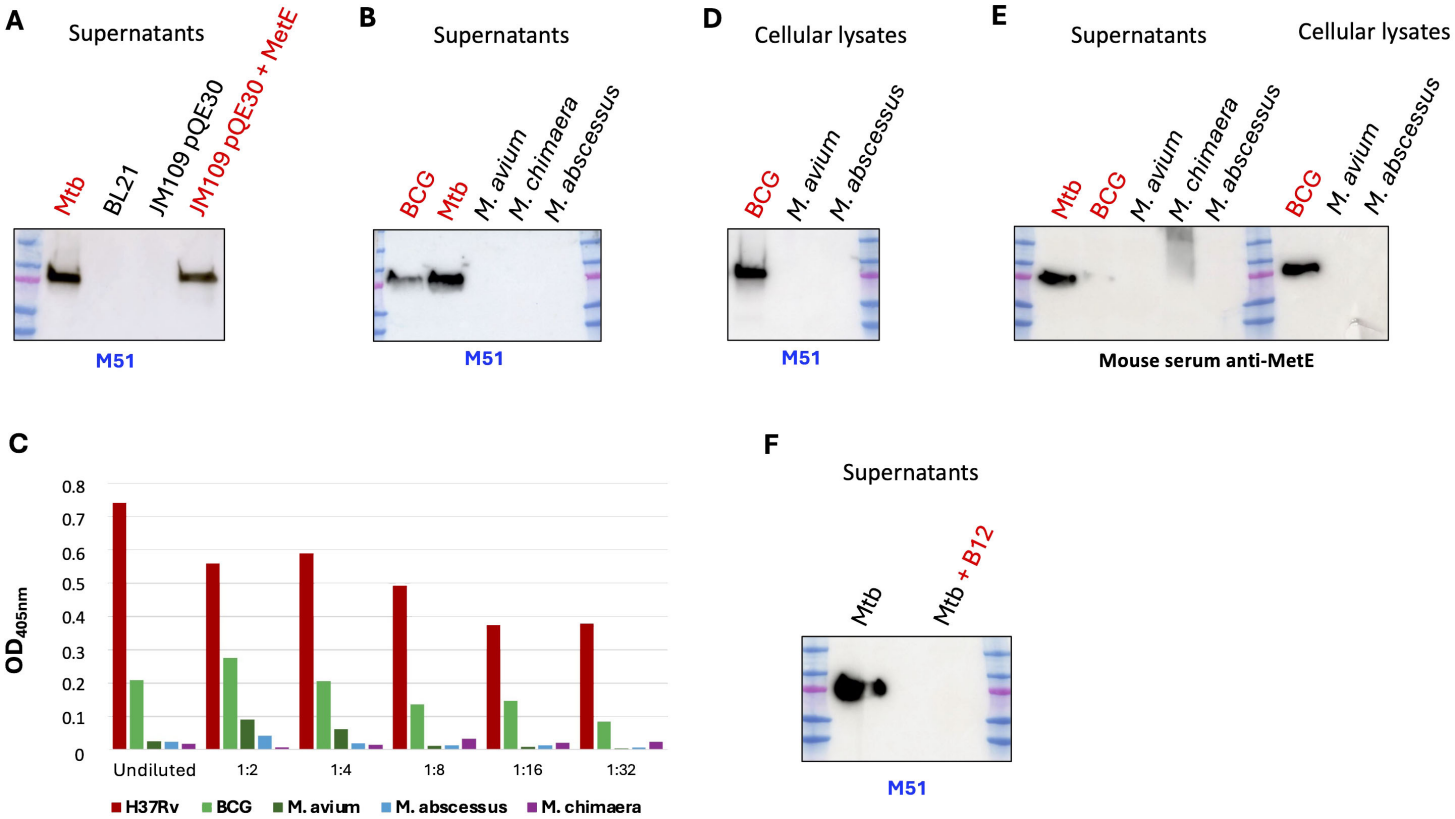
Analysis of the MetE presence among mycobacteria. WB performed on culture supernatants **(A, B)** or on bacterial pellet lysates **(D)**, by using mAb M51 as primary antibody. One experiment representative of two experiments is shown. **(C)** Binding of M51 to MetE by using serial dilutions of bacterial supernatant-coated ELISA plates. One experiment representative of two experiments is shown. **(E)** SDS-PAGE of supernatants and bacterial lysates followed by WB using rMetE immunized mouse polyclonal serum. **(F)** SDS-PAGE of Mtb supernatant with (lane 4) and without (lane 2) added vitamin B12 (at 10 µg/mL) followed by WB using M51. Lane 3, empty; one experiment representative of three experiments is shown.

MetE protein, as a pivotal part of the methionine metabolic pathway, is ubiquitous among bacteria; moreover, it is a major cellular component in wild-type *E. coli* grown in minimal medium, where it is estimated to be 3%–5% of the soluble proteins (17, 18). We showed that mAb M51 binds the N-terminal region of MetE, which, despite being the more dissimilar region of the protein, still shares 38% amino acid sequence identity with *E. coli* (**Supplementary Figure S3A**). To test whether mAb M51 cross-reacts with MetE of *E. coli*, we performed WB analysis on the culture supernatants of *E. coli* grown in minimal medium. In **Figure 2A**, it is possible to observe that mAb M51 does not bind *E. coli* BL21, a bacterium that was previously shown to produce abundant MetE protein (19) or *E. coli* JM109 containing the empty pQE30 vector. Moreover, the same mAb does not bind proteins in supernatants or cellular lysates of *S. pneumoniae, S. aureus*, or *A. baumanii*, bacteria showing the same MetE sequence homology of *E. coli* (**Supplementary Figure S5**). On the other hand, mAb M51recognizes MetE in the supernatant of Mtb, BCG, and *E. coli* JM109, which contains the expression vector pQE30-MetE. In addition, mAb M51 does not recognize the *E. coli* BL21 cellular lysate (data not shown), excluding the possibility that mAb M51 fails in recognizing MetE in the supernatants because *E. coli* is unable to secrete it. The alignments of MetE sequences among mycobacteria, performed by Clustal Omega software tool, revealed high sequence identity between H37Rv, BCG, and NTM (**Supplementary Figure S3B**). While H37Rv and BCG MetE sequences are 100% identical, the sequences of NTM MetE are different, but still with high identity. MetE sequences in *M. avium* and *M. chimaera* share 86% amino acid identity with H37Rv, while MetE of *M. abscessus* is 79% identical to H37Rv. Despite great sequence homology, we surprisingly observed, by WB analysis (**Figures 2B, D**), that mAb M51 is able to bind MetE protein in the culture supernatants and in bacterial lysates of Mtb and BCG, but not of NTM. ELISA analysis performed with the same culture supernatants as the coating antigen confirms that M51 can recognize MetE only in the supernatant of Mtb and, although at a lower extent, of BCG (**Figure 2C**).

We finally performed WB analysis on the same culture supernatants and bacterial lysates by using the serum of a mouse immunized with rMetE, and also in this case, the NTM supernatants and bacterial lysates were negative for the presence of MetE protein (**Figure 2E**): the absence of a positive signal for MetE in NTM strains, with both mAb M51 and polyclonal antibody anti-MetE, may indicate that the NTM are unable to express MetE protein, unlike Mtb and BCG.

MetE is a cobalamine-independent methionine synthase since it does not require vitamin B12 as a cofactor; moreover, as previously demonstrated (20), the expression of *metE* gene is negatively regulated by vitamin B12. To confirm the ability of vitamin B12 to repress *metE* expression, we performed a WB on the supernatant of H37Rv Mtb grown in 7H9 medium in the presence or absence of vitamin B12. As shown in **Figure 2F**, the addition of 10 μg/mL of B12 to the growth medium causes the absence of MetE protein in the Mtb supernatant.

### 3.6 MetE is expressed and secreted by actively replicating, but not dormant Mtb

A typical feature of Mtb is its ability to enter in a state of dormancy when it encounters limiting growth conditions, such as oxygen depletion and/or nutrient starvation. In this condition, it slows its metabolic activities down, stops to replicate, and survives until favorable conditions are repristinated. *In vitro*, these non-replicating dormant bacilli can be obtained and studied by setting up the hypoxia-induced dormancy Wayne model (9, 21), which consists of culturing Mtb in tightly closed tubes in which oxygen concentration becomes gradually limited, determining growth arrest and dormancy. After an initial log phase, Mtb stopped growing at day 7, entering a non-replicating persistence stage 1 (NRP-1); after day 11, highly hypoxic conditions are reached (stage NRP-2). Around day 25, late dormancy phase is established, after enduring hypoxic response activation (22).

**Figure 3:**
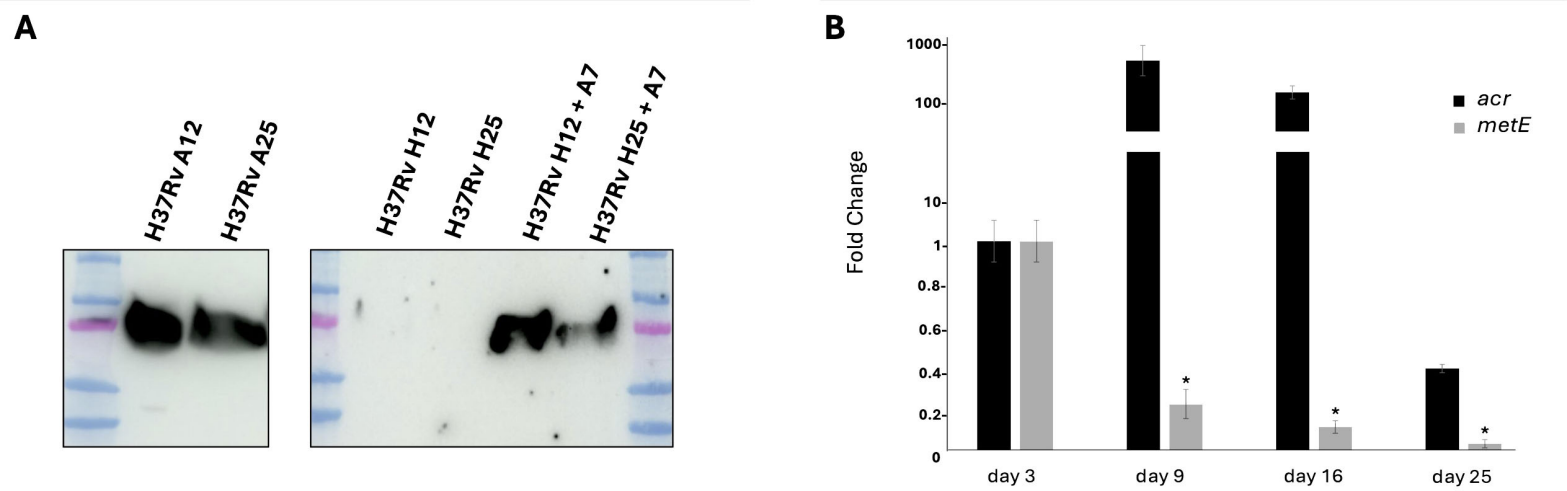
Expression of MetE in actively replicating and dormant Mtb. **(A)** WB performed with mAb M51 on supernatants of aerobic culture collected after 12 and 25 days of growth (A12 and A25), anaerobic non-replicating culture (H12 and H25) and reactivated anaerobic culture after 7 days of reaeration (H12+A7 and H25+A7). **(B)** qRT-PCR analysis of the *metE* and *acr* gene expression after 3, 9, 16, and 25 days of dormancy. All data are expressed as fold change compared to day 3 of *metE* or *acr*, respectively; error bars indicate the standard deviations of technical triplicates and (*) indicates *p*-values < 0.01 compared to control at day 3. One experiment representative of two experiments is shown.

In order to study the expression of MetE during the different stages of Mtb, from aerobic, actively replicating to anaerobic, non-replicating bacilli, we investigated the presence of MetE protein in the supernatants of dormant Mtb cultures (H) and compared them with those of actively replicating cultures (A). Hypoxic dormant culture supernatants were collected after 12 (H12) and 25 (H25) days of growth, to mimic non-replicating persistence stage 2 (NRP-2) and late dormant bacilli, respectively. WB performed with mAb M51 clearly demonstrated that MetE protein is not detectable in the supernatant of dormant hypoxic Mtb H12 and H25 (**Figure 3A**). When dormant Mtb cultures were resuscitated from their non-replicating state, by reaeration of anaerobic cultures and further 7 days of aerobic growth (H12+A7 and H25+A7), we observed the restoration of the MetE detection in the culture supernatants (**Figure 3A**). To better understand the expression of MetE in dormant Mtb, we analyzed, by qRT-PCR, the transcription of *metE* gene at different stages of dormancy: in **Figure 3B**, we observed that while dormant bacilli showed the expected upregulation of the typical dormancy gene alpha-crystallin (*acr*) (23), *metE* gene was clearly downregulated in 9- to 25-day-old hypoxic cultures. The absence of MetE protein observed in the supernatant of dormant Mtb reflects the low level of *metE* mRNA observed in Mtb grown under hypoxic conditions.

### 3.7 MetE as new potential immunological biomarker: preliminary proof of concept

Despite the fact that serological tests have been considered attractive for the diagnosis of active TB, the majority of those on market are not validated or recommended for use in the general population. Several studies based on serum antibodies against ESAT-6 and CFP-10 failed to demonstrate an ability to discriminate between active TB and latent infection (24).

**Figure 4:**
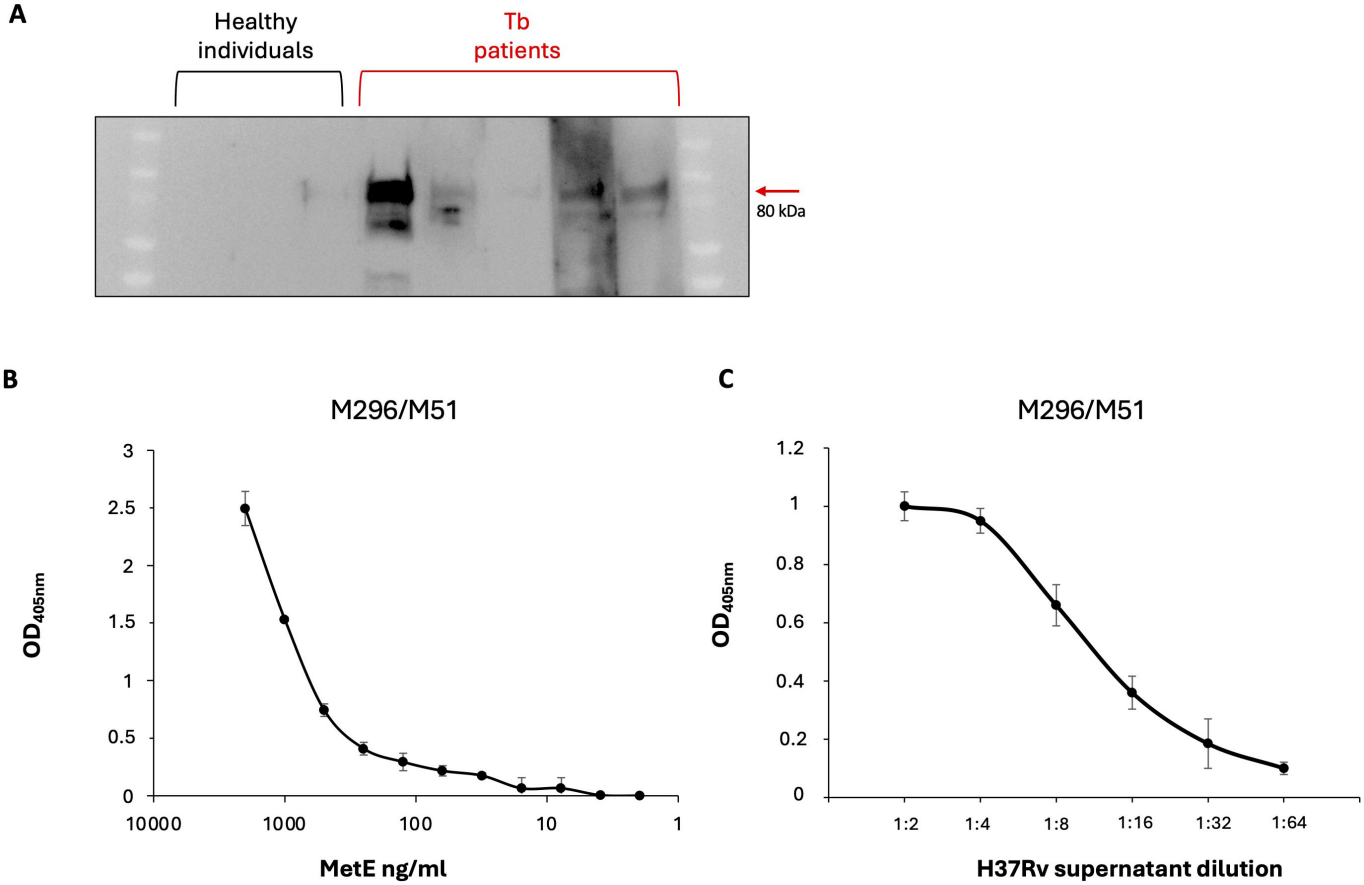
Preliminary proof of concept for diagnostics. **(A)** SDS-PAGE of rMetE followed by WB using sera from three different healthy individuals or five independent TB patients as primary antibodies. One experiment representative of two experiments is shown. **(B)** Sandwich ELISA performed with the M296/M51 pair of mAbs for the detection of scalar amounts of recombinant MetE or of **(C)** the presence of antigen in serial dilutions of H37Rv culture supernatants. Error bars indicate standard deviations of technical triplicates. One experiment representative of three experiments is shown.

As a preliminary proof of concept, we evaluated the potential efficacy of a serological test to identify Mtb-infected individuals by detecting MetE-specific antibodies. **Figure 4A** shows that serum antibodies from patients with active TB, but not those from healthy individuals, bind rMetE, giving positive signals in WB. One of the unmet needs in the screening for Mtb infection is the possibility to differentiate patients with active disease from individuals with LTBI. Since MetE is a protein that should be secreted during active replication of Mtb, and not in the dormant phase, the mAbs that we have isolated could be potentially used to detect the release of this Mtb-specific antigen in biofluids as a marker of active TB. We performed a sandwich ELISA with different pairs of our mAbs in order to identify the best one for measuring MetE protein. The mAb M296 (IgG2b subclass) as coating antibody and mAb M51 (IgG1 subclass) as secondary antibody were chosen since they recognize different epitopes and were the couple of mAbs that give the best results in terms of sensitivity of the test. We showed that this mAbs combination permitted the detection of very low quantity of both the rMetE protein (**Figure 4B**) and the wild-type MetE secreted in the culture supernatant of Mtb H37Rv (**Figure 4C**).

## 4 Discussion

In this work, we immunized mice with an Mtb culture supernatant to isolate new mAbs specific for Mtb proteins that could be interesting to better characterize Mtb pathogenicity and for the possible preparation of new immunodiagnostics.

Among many generated mAbs, we focused on those recognizing the same antigen of approximately 80 kDa, which was identified, by mass spectrometry, as the Rv1133c, the MetE protein.

The presence of MetE protein is ubiquitous in bacteria and its amino acid sequence is highly conserved, suggesting an essential role of this enzyme in bacterial metabolism.

For example, MetE enzyme is very abundant in *E. coli* growing aerobically in minimal medium, where it represents approximately 3% to 5% of the entire protein content. This could be explained by the fact that MetE is approximately 50 times less active than MetH, a cobalamin-dependent methionine synthase, the other enzyme involved in methionine synthesis, which use vitamin B12 as a cofactor to produce methionine (17, 25). Both MetH and MetE exist in bacteria, but animals only possess cobalamin-dependent methionine synthase while fungi and plants only utilize cobalamin-independent methionine synthase (26).

Sequence alignment of Mtb MetE revealed 100% identity with BCG, 48% amino acid identity with *E. coli*, and an amino acid identity of 50%, 44%, and 50% with *S. pneumoniae, S. aureus*, and *A. baumanii*, respectively. Moreover, the sequence alignment of Mtb MetE revealed 79%–86% identity with non-tuberculous mycobacteria, particularly 79% for *M. abscessus* and 86% for *M. avium* and *M. chimaera* (**Supplementary Figure S3A, B**).

Despite this similarity, WB analysis showed that mAb M51 binds to MetE only in the supernatants or cell lysates of Mtb and BCG, but not in those of *E. coli* (**Figure 2A**) or NTM (**Figures 2B, D**) or those of *S. pneumoniae, S. aureus*, and *A. baumanii* (**Supplementary Figure S5A**), suggesting a high specificity of this mAb for an Mtb antigen.

Comparison of the amino acid sequence between Mtb and *E. coli* MetE indicated a higher diversity in the N-terminal region of the proteins, where mAb M51 recognizes its epitope, and this may explain its selectivity in binding Mtb, but not *E. coli* MetE.

Finding a reason for the inability of mAb M51 to bind MetE from NTM is difficult. The amino acid sequence of MetE revealed that Mtb and BCG differ from NTM in the first seven amino acids, and a putative signal peptide can be identified by using the SignalIP 5.0 software (27, 28). To test whether the lack of MetE recognition by our mAb M51 in the NTM supernatants was due to their inability to secrete MetE, we performed a WB on bacterial pellet lysates. Results show that mAb M51 recognizes MetE in Mtb or BCG lysates, but not in the bacterial pellet lysate of NTM (**Figure 2D**). The same result was obtained using a polyclonal serum of a mouse immunized with rMetE (**Figure 2E**). In conclusion, owing to the high MetE sequence identity between Mtb and NTM, the possibility that MetE is not produced in cultures of NTM is envisaged. In fact, *metE* gene transcription is regulated by the presence of a riboswitch upstream the promoter region that represses MetE expression upon binding of vitamin B12 (20, 29). We confirmed that vitamin B12 supplementation effectively represses MetE expression in Mtb and makes it undetectable in the culture supernatant (**Figure 2F**). It has been previously demonstrated that while NTM are able to produce cobalamin, Mtb lost this ability. Analysis of mycobacteria genomes revealed the deletion of the *cobF* gene locus, encoding for a key component of the cobalamin biosynthetic pathways, in the species of Mtb complex, while it is normally present in NTM. The evolution of intracellular parasitic lifestyle of Mtb could be in part due to the deletion of the *cobF* gene and the consequent need to uptake vitamin B12 from the host (29–31). Therefore, the sensitivity to B12 could be a possible reason for the abundant presence of MetE protein in the Mtb supernatant and lysates and for its absence in NTM supernatant and lysates: the lack of B12 in Mtb determines the inactivation of MetH (the absence of the B12 cofactor) and the overexpression of MetE protein (the lack of the B12 repressor) to cover for the entire production of methionine, while the production of B12 in NTM could switch off MetE production, making our mAb M51 unable to detect it in supernatants and in NTM pellets.

One of the major obstacles in controlling TB is the lack of a rapid but efficient diagnostic tool able to differentiate active TB from LTBI.

Traditional TB diagnostic methods, such as culture or smear microscopy, are slow or low in sensitivity. Molecular techniques, such as GeneXpert MTB/RIF, are costly and often unavailable in primary-care settings because of their infrastructure needs (32). The tuberculin skin test (TST) and the interferon gamma release assay (IGRA) have low ability to discriminate active from latent TB (33–35) and these tests cannot be used to predict whether an individual with LTBI will develop active TB or whether therapy for LTBI could be effective to decrease the risk of developing active TB (36). Moreover, while the diagnosis of TB is relatively easy in lung TB, that permits the Mtb cultures and/or the use of molecular methods to identify Mtb genes in sputa or bronchoalveolar lavages, the diagnosis of non-pulmonary TB is more complex and often requires interventional radiology to collect samples.

Our data and previous ones (28, 37) indicated MetE as one of the most secreted proteins in Mtb cultures, suggesting that also actively replicating Mtb in TB produces and releases MetE by active secretion or after mycobacterial cell lysis. Moreover, among the hundreds of proteins detected in Mtb cultures, only MetE induced a specific immune response that allowed the isolation of mAbs in different mice immunized with the same culture supernatant, suggesting its high immunogenicity. Interestingly, in a previous work, Hadizadeh et al. isolated antibodies against Rv1133c, among other proteins, from TB patients’ sera (38). Therefore, we assume that a robust immune response against MetE is generated upon Mtb infection. This hypothesis was firstly tested using recombinant MetE to analyze a small set of sera from TB patients by WB analysis. Results showed anti-MetE antibodies in sera of TB patients, but not in sera of healthy controls. Measurement of antibodies specific for Mtb antigens has limited chances to contribute significantly to the diagnosis of TB and its stages in clinical settings (1, 39, 40). However, if the high response to MetE will be confirmed in larger samples of patients with TB or LTBI, the measurement of antibodies specific for the N-terminal region of MetE could contribute to identify individuals with previous contacts with Mtb or BCG in epidemiologic surveys.

Biomarkers of TB detectable in biological samples are assumed to be useful to rapidly identify patients with active TB. The most studied nonsputum biomarkers are LAM (41), culture filtrate proteins, and early secretory antigenic target-6 (42, 43). However, because of the Mtb compartmentalization *in vivo* and their release or secretion in low amounts, these biomarkers tend to be hardly detectable, have low clinical sensitivity, and are non-specific for Mtb, due to similarities with biomarkers of other mycobacteria (42).

Interestingly, by using the well-established hypoxia-induced dormancy Wayne model, we analyzed the production of MetE protein during the different stages of growth, and we demonstrated the absence of this antigen in the culture supernatants of non-replicating dormant Mtb (**Figure 3A**), as well as the downregulation of *metE* gene in dormant bacilli (**Figure 3B**). MetE protein was detectable in the supernatant of active-replicating Mtb and, in the supernatants of resuscitated bacilli, after recovering from dormancy. These results lead us to hypothesize that detection of MetE antigen could be potentially used to discriminate patients with active TB, in which MetE could be measured, from individuals with LTBI, in which MetE could not be detected. To this end, we explored the possibility to use our mAbs to set up an antigenic test to measure MetE antigen in biological fluids. We tested several pairs of our mAbs in a sandwich ELISA and identified in the M296/M51 pair the best one to detect both the recombinant and Mtb-secreted MetE (**Figure 4B**). The performance of the test was extremely good, permitting us to measure very low amounts of MetE. In this context, considering that MetE is released in high amounts in Mtb cultures and reasonably also in biofluids, if this novel biomarker of TB were detectable in biological samples, it would be useful, in addition to IGRA, to rapidly identify patients with active TB or with LTBI and to predict those at higher risk of developing active disease. In fact, IGRA values denote the expression of the T-cell response to Mtb antigens, and even if they are not influenced by BCG vaccination, positivity to an IGRA test is obtained also in individuals with LTBI who maintain a memory T-cell response against Mtb antigens. Therefore, additional clinical examinations are required to identify patients requiring specific treatment for active TB. MetE was shown to be one of the more released antigens in Mtb cultures, and since MetE is not released by dormant bacteria, we hypothesize that only patients with active TB in whom Mtb actively replicates may display MetE in their biofluids. The measure of MetE in IGRA-positive individuals would be a marker of active TB. Our preliminary *in vitro* data are encouraging for further studies on the field that we are planning to initiate.

In conclusion, the isolation of mAbs specific for MetE highlighted the importance of this enzyme in the host immune response and allowed us to follow its secretion in different stages of Mtb growth. Finally, the identification of the N-terminal fragment of MetE as Mtb specific, the readiness of its recombinant form, together with the availability of high-affinity mAbs specific for Mtb MetE set the stage for the development of new diagnostic tools to be tested in the field.

## Data Availability

The raw data supporting the conclusions of this article will be made available by the authors, without undue reservation.
