# Supplementary material for: Identification of Rv1133c (MetE) as a marker of Mycobacterium tuberculosis replication and as a highly immunogenic antigen with potential immunodiagnostic power
Source: Front Immunol. 2024 Oct 4;15:1464923. doi: 10.3389/fimmu.2024.1464923 (PMC11486704; doi:10.3389/fimmu.2024.1464923)
Supplement: Supplementary file 1 [file DataSheet1.pdf]

Supplementary Material

1 Supplementary Figures and Tables

1.1 Supplementary Figures

Supplementary Figure S1: Scheme of the strategy followed to obtain monoclonal antibodies (MoAb) specific for released Mtb proteins. Sterile culture medium supernatants (SCMS); Immunization of mice with SCMS, production of MoAbs specific for SCMS components. Selection of MoAbs binding SCMS and recognizing Mtb cells analyzed by immunofluorescence (IF) by flow cytometetry. Immunoprecipitation and characterization of antigens recognized by MoAbs by LC MALDI.

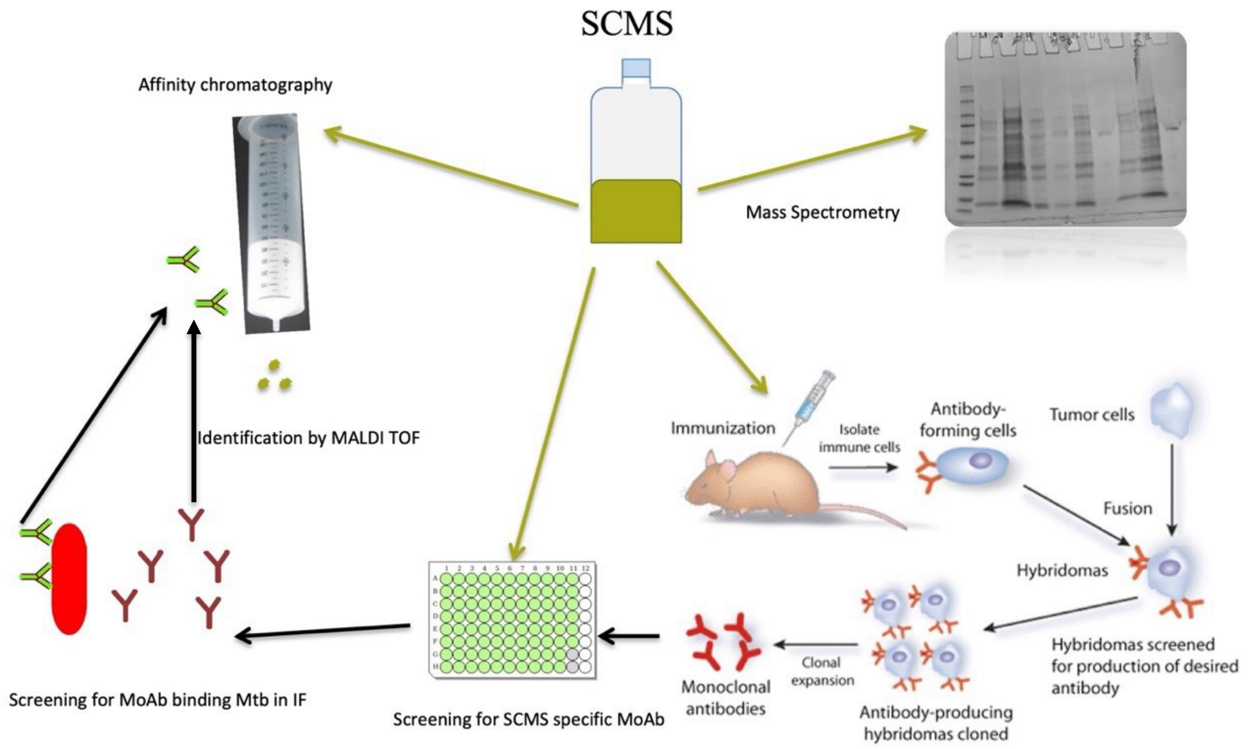

Supplementary Figure S2. **Immunoprecipitation of identified antigen. a)** WB analysis after SDS-PAGE of H37Rv SCMS by using mAb M35 as primary antibody. **b)** SDS-PAGE of isolated mAbs and (M35 and M29) and immuno-precipitation complex of protein (from the SCMS) and purified mAbs (M35 + protein and M29 + protein). Red arrows indicate the 80 kDa protein identified.

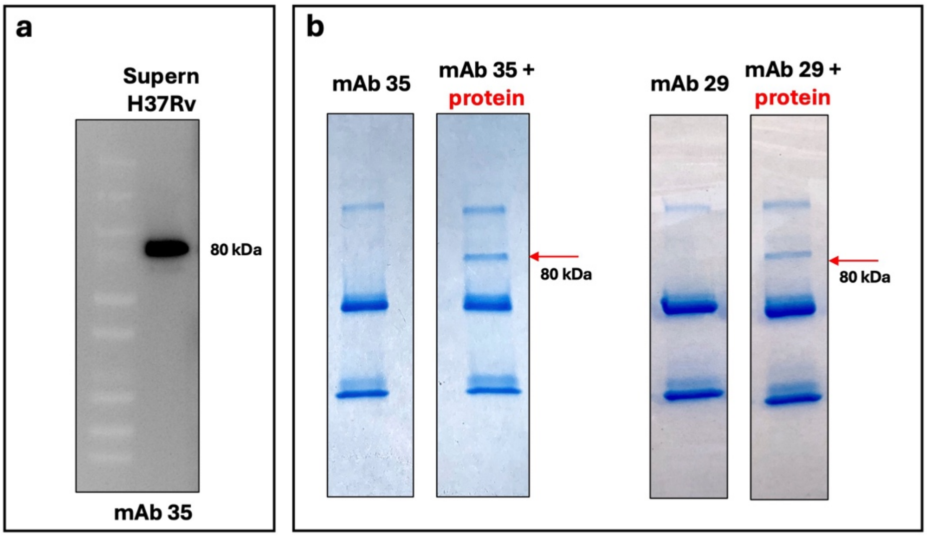

Supplementary Figure S3. A) MetE sequence comparison between Mtb H37Rv (query) and *E. coli* BL21 (subject), performed by BLASTP NCBI tool. The full length (aa 1-759) comparison shows amino acid sequence identity of 48%, while comparison of the N-terminal region (aa 1-420), reveal 38% sequence identities. (+) indicates functional equivalence, amino acid not identical but with similar characteristics. **B)** Clustal Omega multiple sequence alignment. Amminoacidic sequence of Mtb H37Rv Rv1133c/MetE protein (NCBI accession number CCP43887.1) was compared to that of BCG (NCBI accession number A1KHS4), *M. abscessus* (NCBI accession number WP_005112521), *M. avium* (NCBI accession number WP_009975418) and *M. chimaera* (NCBI accession number AOS91244). (*) indicates conserved amminoacid; (:) indicates the alignment has strongly similar residues; (.) indicates the residues have weakly similar property.

A

```
Query  16   GSPRIGPRRELKRATEGYWAGRTSRSELEAVAATLRRDTWSALAAAGLDSVPVNTFSYYD  75
            G PR+G RRELK+A E YWAG ++R EL AV   LR   W     AG+D +PV  F++YD
Sbjct  9    GFPRVGLRRELKKAQESYWAGNSTREELLAVGRELRARHWDQQKQAGIDLLPVGDFAWYD  68

Query  76   QMLDTAVLLGALPPRVSPVSDG---LDRYFAAARGT----DQIAPLEMTKWFDTNYHYLV  128
             +L T++LLG +PPR     DG   +D  F   RG     +  A  EMTKWF+TNYHY+V
Sbjct  69   HVLTTSLLLGNVPPR-HQNKDGSVDIDTLFRIGRGRAPTGEPAAAAEMTKWFNTNYHYMV  127

Query  129  PEIGPSTTFTLHPGKVLAELKEALGQGIPARPVIIGPITFLLLSKAVDGAGAPIERLEEL  188
            PE      F L    ++L E+ EAL  G   +PV++GP+T+L L K        +  L ++
Sbjct  128  PEFVKGQQFKLTWTQLLEEVDEALALGHKVKPVLLGPVTWLWLGKVKGEQFDRLSLLNDI  187

Query  189  VPVYSELLSLLADGGAQWVQFDEPALVTDLSPDAPALAEAVYTALCSVSNRPAIYVATYF  248
            +PVY ++L+ LA  G +WVQ DEPALV +L     A  +A   A  ++  +  + + TYF
Sbjct  188  LPVYQQVLAELAKRGIEWVQIDEPALVLELPQ---AWLDAYKPAYDALQGQVKLLLTTYF  244

Query  249  GDPGAALPALARTPVEAIGVDLVAGADTSVAGVPELAGKTLVA-GVVDGRNVWRTDLEAA  307
                  L  +   PV+ + VDLV G D        L    L++ G+++GRNVWR DL
Sbjct  245  EGVTPNLDTITALPVQGLHVDLVHGKDDVAELHKRLPSDWLLSVGLINGRNVWRADLTEK  304

Query  308  LGTLATLLGSAATVAVSTSCSTLHVPYSLEPETDLDDALRSWLAFGAEKVREVVVLARAL  367
               +  ++G    + V++SCS LH P  L  ET LD  ++SW AF  +K  E+ +L  AL
Sbjct  305  YAQIKDIVGKR-DLWVASSCSLLHSPIDLSVETRLDAEVKSWFAFALQKCHELALLRDAL  363

Query  368  RDGHDAVADEIASSRAAIASRKRDPRLHNGQIRARIEAIVASGAHRGNAAQRRA-SQDAR  426
              G  A     +A   A I +R+   R+HN  +  R+ AI A  + R N  + RA +Q AR
Sbjct  364  NSGDTAA---LAEWSAPIQARRHSTRVHNPAVEKRLAAITAQDSQRANVYEVRAEAQRAR  420

Query  427  LHLPPLPTTTIGSYPQTSAIRVARAALRAGEIDEAEYVRRMRQEITEVIALQERLGLDVL  486
              LP  PTTTIGS+PQT+ IR  R   + G +D   Y   + + I + I  QERLGLDVL
Sbjct  421  FKLPAWPTTTIGSFPQTTEIRTLRLDFKKGNLDANNYRTGIAEHIKQAIVEQERLGLDVL  480

Query  487  VHGEPERNDMVQYFAEQLAGFFATQNGWVQSYGSRCVRPPILYGDVSRPRAMTVEWITYA  546
            VHGE ERNDMV+YF E L GF  TQNGWVQSYGSRCV+PPI+ GDVSRP  +TVEW  YA
Sbjct  481  VHGEAERNDMVEYFGEHLDGFVFTQNGWVQSYGSRCVKPPIVIGDVSRPAPITVEWAKYA  540

Query  547  QSLTDKPVKGMLTGPVTILAWSFVRDDQPLADTANQVALAIRDETVDLQSAGIAVIQVDE  606
            QSLTDKPVKGMLTGPVTIL WSF R+D      A Q+ALA+RDE  DL++AGI +IQ+DE
Sbjct  541  QSLTDKPVKGMLTGPVTILCWSFPREDVSRETIAKQIALALRDEVADLEAAGIGIIQIDE  600

Query  607  PALRELLPLRRADQAEYLRWAVGAFRLATSGVSDATQIHTHLCYSEFGEVIGAIADLDAD  666
            PALRE LPLRR+D   YL+W V AFR+   +  D TQIHTH+CY EF +++ +IA LDAD
Sbjct  601  PALREGLPLRRSDWDAYLQWGVEAFRINAAVAKDDTQIHTHMCYCEFNDIMDSIAALDAD  660

Query  667  VTSIEAARSHMEVLDDLNAIGFANGVGPGVYDIHSPRVPSAEEMADSLRAALRAVPAERL  726
            V +IE  RS ME+L+      + N +GPGVYDIHSP VPS E +   L+ A + +PAERL
Sbjct  661  VITIETSRSDMELLESFEEFDYPNEIGPGVYDIHSPNVPSVEWIEALLKKAAKRIPAERL  720

Query  727  WVNPDCGLKTRNVDEVTASLHNMVAAAREVRAG  759
            WVNPDCGLKTR   E  A+L NMV AA+ +R G
Sbjct  721  WVNPDCGLKTRGWPETRAALANMVQAAQNLRRG  753
```

B

```
M.abscessus         -------qplkattlgsarigprrelkratesywagrtsreeletvaaglrrdnwtalaa    53
M.tuberculosis      mtqpvrrqpftatitgsprigprrelkrategywagrtsrseleavaatlrrdtwsalaa    60
BCG                 mtqpvrrqpftatitgsprigprrelkrategywagrtsrseleavaatlrrdtwsalaa    60
M.avium             -------qaftatvvgsprigpkrelkrategywagrtgraelekvaatlrrdtwaglaa    53
M.chimaera          -------qpftatvtgsprigpkrelkrategywagrtsrselesvaatlrrdtwaslaa    53
                           * :.**  ** ****:********.******.* *** *** ****.*:.***

M.abscessus         agldsvpvntfsyydqvldtavllgalpprvsgiaddldryfaaargnadvtplemtkwf    113
M.tuberculosis      agldsvpvntfsyydqmldtavllgalpprvspvsdgldryfaaargtdqiaplemtkwf    120
BCG                 agldsvpvntfsyydqmldtavllgalpprvspvsdgldryfaaargtdqiaplemtkwf    120
M.avium             agldsvpvntfsyydqmldtavmldalperarqvsddldryfaaargnsdvaplemtkwf    113
M.chimaera          agldsvpvntfsyydqmldtavmldalparatqvsddldryfaaargnsdvaplemtkwf    113
                    ****************:*****:*.*** *.  ::*.**********. :::********

M.abscessus         dtnyhylvpeigpdttfelnpaklfgelkeaqaldiparpvvvgpitflalsksvdgaga    173
M.tuberculosis      dtnyhylvpeigpsttftlhpgkvlaelkealgqgiparpviigpitflllskavdgaga    180
BCG                 dtnyhylvpeigpsttftlhpgkvlaelkealgqgiparpviigpitflllskavdgaga    180
M.avium             dtnyhyivpeiapttkfalnpdkvlsdlkealaqgiparpvvigpitflllskgvdgaga    173
M.chimaera          dtnyhyivpeiapatkfalnpdkvlselkealaqgiparpvvigpitflllskgvdgaga    173
                    ******:****.* *.* *:* *::.:**** .  ******::****** ***.******

M.abscessus         pierldevvalyeqllvqlaeagvgwvqidepvlvtdilpngpelaervygrlgtvadrp    233
M.tuberculosis      pierleelvpvysellslladggaqwvqfdepalvtdlspdapalaeavytalcsvsnrp    240
BCG                 pierleelvpvysellslladggaqwvqfdepalvtdlspdapalaeavytalcsvsnrp    240
M.avium             pierleelvpiyaellslladngaqwvqldepalvtdmcadapalaeavynklgsasnrp    233
M.chimaera          pierleelvgiysellslladngaqwvqldepalvtdaspdapalaeavynklgavsnrp    233
                    *****:*:* :* :**  **: *. ***:***.****   :.* *** **  * :.::**

M.abscessus         ailvasyfgelgaalpalartpieaiavdlvygsasavasvpelagktlvagvvdgrniw    293
M.tuberculosis      aiyvatyfgdpgaalpalartpveaigvdlvagadtsvagvpelagktlvagvvdgrnvw    300
BCG                 aiyvatyfgdpgaalpalartpveaigvdlvagadtsvagvpelagktlvagvvdgrnvw    300
M.avium             aiyvatyfgdpgaslaglartpveaigvdlvygpdtavaavpelagktvvagvvdgrnvw    293
M.chimaera          siyvatyfgdpgaalaglartpveaigvdlvygpdtavaavpeladkvlvagvvdgrniw    293
                    :* **:***: **:* .*****:***.**** *  ::**.*****.*.:*********:*

M.abscessus         rtnlqsalstlasllgsaesvavstscstlhvpyslepeteldddqlrswlafadekvkev    353
M.tuberculosis      rtdleaalgtlatllgsaatvavstscstlhvpyslepetdlddalrswlafgaekvrev    360
BCG                 rtdleaalgtlatllgsaatvavstscstlhvpyslepetdlddalrswlafgaekvrev    360
M.avium             rtdlaaaldklttllgsaarvavstscstlhvpyslepetdlddalrswlafgrekvaev    353
M.chimaera          rtdlqaaldklasllgsaaavavstscstlhvpyslepetgldealrswlafgqekvgev    353
                    **:* :**..*::*****  ******************** **: *******. *** **

M.abscessus         vvlaralsegreavadeiaasnaavesrktdprlnngqirtrldsilaagvsrgdaaerr    413
M.tuberculosis      vvlaralrdghdavadeiassraaiasrkrdprlhngqirarieaivasgahrgnaaqrr    420
BCG                 vvlaralrdghdavadeiassraaiasrkrdprlhngqirarieaivasgahrgnaaqrr    420
M.avium             vtlaralrdgreavadeiaasnaavasrksdprlhndrvraridsivasgshrgdpaqrr    413
M.chimaera          valaralregreavadeiaasnaavasrksdprlhndriraridsivasgahrgdaarrr    413
                    *.***** :*::*******:*.**: *** ****:*.::*:*:::*:*:*  **: *.**

M.abscessus         rsqderlnlpelptttigsfpqtveirkarqalakgeiddaeyvrqmraevadvialqek    473
M.tuberculosis      asqdarlhlpplptttigsypqtsairvaraalrageideaeyvrrmrqeitevialqer    480
BCG                 asqdarlhlpplptttigsypqtsairvaraalrageideaeyvrrmrqeitevialqer    480
M.avium             asqdarlhlpplptttigsypqtsairkaraalrsgeidqaeyerrmkkeiadvitlqeq    473
M.chimaera          asqderlqlpplptttigsfpqtaqirkaraalvageidaaeyekrmkqeiadviklqen    473
                     *** **:** ********:***  ** ** **  **** *** ::*: *:::** ***.
```

```
M.abscessus         lgldvlvhgeperndmvqyfaeqldgffatqngwvqsygsrcvrppilygdvarqqpmtv     533
M.tuberculosis      lgldvlvhgeperndmvqyfaeqlagffatqngwvqsygsrcvrppilygdvsrpramtv     540
BCG                 lgldvlvhgeperndmvqyfaeqlagffatqngwvqsygsrcvrppilygdvsrpramtv     540
M.avium             lgldvlvhgeperndmvqyfaeqlegffatqngwvqsygsrcvrppilygdvvrthpmtv     533
M.chimaera          lgidvlvhgeperndmvqyfaeqlegffatkngwvqsygsrcvrppilygdvirqhpmtv     533
                    **:********************* *****:********************* * : ***

M.abscessus         ewatyaqsltqkhvkgmltgpvtilawsfvrddqplgdtanqialairdetvdlqnagia     593
M.tuberculosis      ewityaqsltdkpvkgmltgpvtilawsfvrddqpladtanqvalairdetvdlqsagia     600
BCG                 ewityaqsltdkpvkgmltgpvtilawsfvrddqpladtanqvalairdetvdlqsagia     600
M.avium             ewityaqsltdkpvkgmltgpvtilawsfvrddqpladtanqvalairdetvdlqaagia     593
M.chimaera          ewakyaqsltdkpvkgmltgpvtilawsfvrddqpladtanqvalairdetvdlqsagia     593
                    ** .******:* ***********************.*****:************ ****

M.abscessus         iiqvdepalrellplrdsekqayldwavgafrlstsgvsdatqihthlcysefgevigai     653
M.tuberculosis      viqvdepalrellplrradqaeylrwavgafrlatsgvsdatqihthlcysefgevigai     660
BCG                 viqvdepalrellplrradqaeylrwavgafrlatsgvsdatqihthlcysefgevigai     660
M.avium             viqvdepalrellplrradqeeylrwavgafrlatsgvadstqihthlcysefgevigai     653
M.chimaera          viqvdepalrellplrradqddylrwavgafrlatsgvadstqihthlcysefgdvigai     653
                    :*************** :::  ** ********:****:*:*************:*****

M.abscessus         adldadvtsieaarshmevlddlnaigfsnsvgpgvydihsprvpsteematslrealka     713
M.tuberculosis      adldadvtsieaarshmevlddlnaigfangvgpgvydihsprvpsaeemadslraalra     720
BCG                 adldadvtsieaarshmevlddlnaigfangvgpgvydihsprvpsaeemadslraalra     720
M.avium             adldadvtsleaarshmevlddlnavgfsnsvgpgvydihsprvpstaeiaeslraalra     713
M.chimaera          adldadvtsieaarshmevlddlnaigfsnsvgpgvydihsprvpsteemaeslraalra     713
                    *********:***************:**:*.***************: *:* *** **:*

M.abscessus         vpaqrlwvnpdcglktrkvdevtsslsnl---------- 742
M.tuberculosis      vpaerlwvnpdcglktrnvdevtaslhnmvaaarevrag 759
BCG                 vpaerlwvnpdcglktrnvdevtaslhnmvaaarevrag 759
M.avium             vpaerlwvnpdcglktrnpdevsaslknmvaaahevrag 752
M.chimaera          vpaerlwvnpdcglktrnsdevtaslknmvaaaqevrag 752
                    ***:*************: ***::** *:
```

Supplementary Figure S4. Cloning and expression of recombinant MetE and $MetE_{420}$ proteins. A) expression vectors pQE30 construction. **B)** SDS-PAGE of recombinant MetE and $MetE_{420}$ with the respective WB analysis performed by using mAb M35.

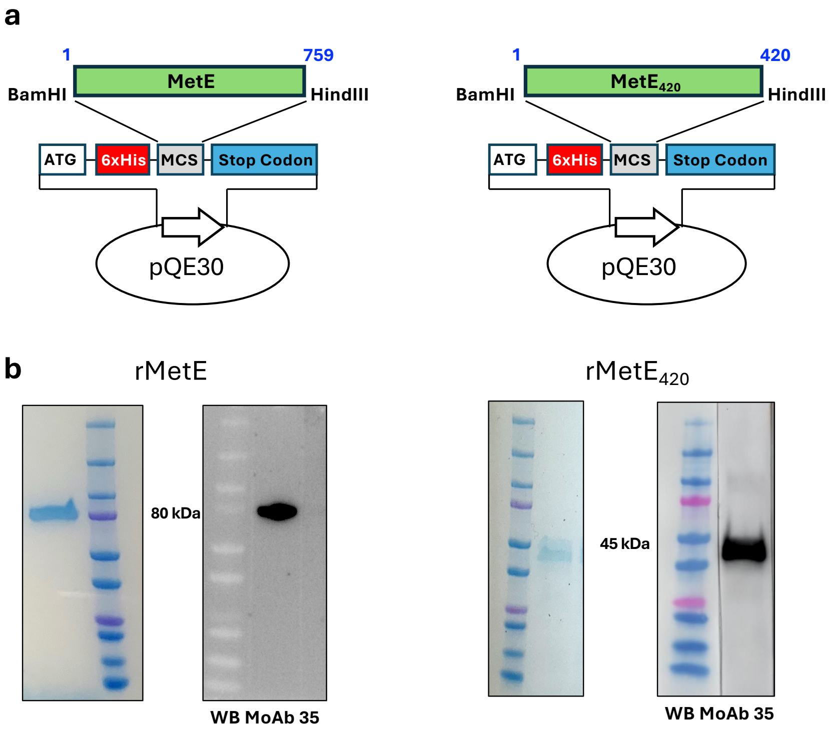

Supplementary Figure S5. WB analysis of the presence of MetE in other interesting bacteria. **A**) SDS-PAGE ponceau colored of culture filtrate supernatant and bacterial pellet lysates, and WB analysis performed with mAb M51. **B**) SDS-PAGE ponceau colored of *S. pneumoniae* bacterial pellet lysates, and WB analysis performed with a mouse serum specific for PspA (pneumococcal surface protein A) as positive control, compared to WB with M51, in order to confirm the good quality of sample.

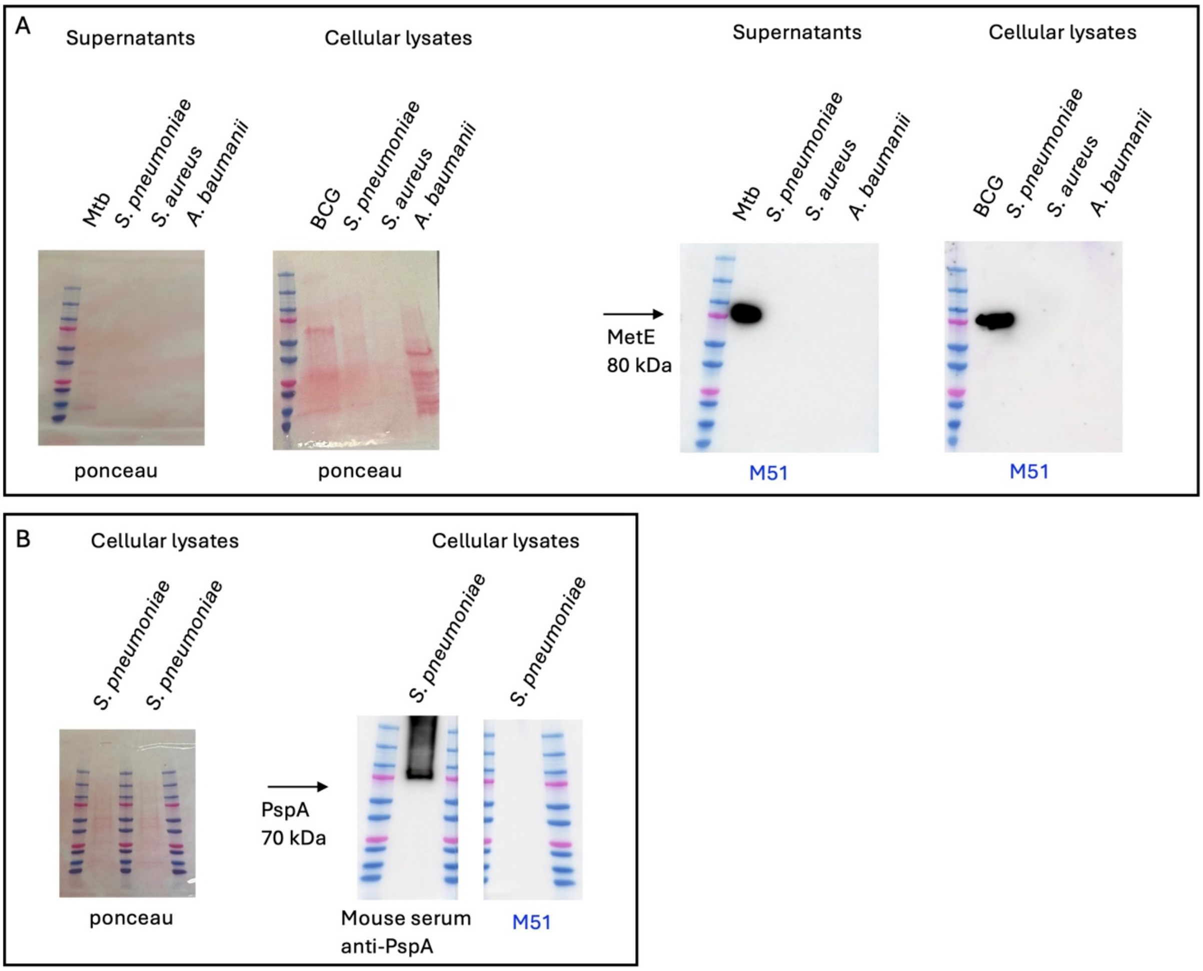

1.2 Supplementary tables

Supplementary table S1. Primers used for cloning and recombinant proteins production and for qRT-PCR analysis.

Primers for cloning	Sequence 5'-3'
**MetEFw**	GGACAGGGATCCACCCAGCCTGTACGTCGTCAACCC
**MetERv**	GCTCGTAAGCTTGCCCGCGCGCACCTCCC
**$MetE_{420}Rv$**	GTCTTGAAGCTTGCGGCGCTGGGCGGCATTG
**Primers for qRT-PCR**	**Sequence 5'-3'**
**16S-S**	GCAGCAGTGGGGAATATTGCACAA
**16S-AS**	TCCACCTACCGTCAATCCGAGAGA
**acr-S**	CCGAGCGCACCGAGCAGAAG
**acr-AS**	GCCTTAATGTCGTCCTCGTCAGCA
**metE2-S**	ACCACGACGATCGGCTCCTA
**metE2-AS**	ACTCGGCCTCGTCGATCTCA

Supplementary table S2. The 10 most significant proteins of Mtb H37Rv growth medium identified through nRP-LC-MS/MS analysis

	Accession		Score	Coverage	# Unique Peptides	# Peptides	MW [kDa]
1	A5U3S7	Rv1908c Catalase-peroxidase OS=Mycobacterium tuberculosis (strain ATCC 25177 / H37Ra) GN=katG PE=3 SV=1 - [KATG_MYCTA]	543.92	54.73	23	23	80.6
2	P9WMK0	Rv2031c Alpha-crystallin OS=Mycobacterium tuberculosis (strain CDC 1551 / Oshkosh) GN=hspX PE=1 SV=1 - [ACR_MYCTO]	492.08	40.28	4	4	16.2
3	P9WN38	Rv2220 Glutamine synthetase 1 OS=Mycobacterium tuberculosis (strain CDC 1551 / Oshkosh) GN=glnA1 PE=3 SV=1 - [GLNA1_MYCTO]	353.32	51.67	12	12	53.5
4	A5U1I0	**Rv1133c 5-methyltetrahydropteroyltri glutamate--homocysteine methyltransferase OS=Mycobacterium tuberculosis (strain ATCC 25177 / H37Ra) GN=metE PE=3 SV=1 - [METE_MYCTA]**	348.74	47.83	20	20	81.5
5	A5U893	Rv3418c 10 kDa chaperonin OS=Mycobacterium tuberculosis (strain ATCC 25177 / H37Ra) GN=groS PE=3 SV=1 - [CH10_MYCTA]	346.47	58.00	5	5	10.8
6	O53166	Rv1475c Aconitate hydratase A OS=Mycobacterium tuberculosis (strain ATCC 25618 / H37Rv) GN=acn PE=1 SV=1 - [ACNA_MYCTU]	316.26	46.55	23	23	102.4
7	P9WNZ4	Rv1094 Putative acyl-[acyl-carrier-protein] desaturase desA2 OS=Mycobacterium tuberculosis (strain CDC 1551 / Oshkosh) GN=desA2 PE=3 SV=1 - [DESA2_MYCTO]	284.47	49.09	9	9	31.3
8	P9WPD4	Rv0896 Citrate synthase 1 OS=Mycobacterium tuberculosis (strain CDC 1551 / Oshkosh) GN=gltA2 PE=3 SV=1 - [CISY1_MYCTO]	279.21	50.58	12	12	47.9
9	P9WGU0	Rv0934 Phosphate-binding protein PstS 1 OS=Mycobacterium tuberculosis (strain CDC 1551 / Oshkosh) GN=pstS1 PE=3 SV=1 - [PSTS1_MYCTO]	256.96	40.64	8	8	38.2
10	A5TYT6	Rv0211 Phosphoenolpyruvate carboxykinase [GTP] OS=Mycobacterium tuberculosis (strain ATCC 25177 / H37Ra) GN=pckG PE=1 SV=1 - [PCKG_MYCTA]	255.54	43.07	16	16	67.2

Supplementary table S3A. LC-MALDI analysis performed on the 80 kDa antigen immuno-precipitated by mAb M35.

	Proteinpilot Unused Score	Proteinpilot Total Score	% of Coverage Sequence	Species	UniProtKB Accession Number
1	**58.27**	**58.27**	**47.8**	**MYCTU**	**P9WK07**
2	35.7	35.7	52.9	MOUSE	P01869
3	12.02	12.02	83	MOUSE	P01837
4	8	8	20.2	HUMAN	P04264
5	7.19	7.19	19.1	BOVIN	P02769
6	6.6	6.6	26.2	MOUSE	P01633
7	4.02	4.02	15.3	HUMAN	P35527
8	4	4	19.7	BOVIN	P34955
9	3.41	3.41	58.5	HUMAN	Q8IUE6
10	3.34	3.34	35.5	DANRE	Q5BJA5
11	2.85	2.85	17	HUMAN	P13645
12	2.6	2.6	33.6	MOUSE	P97430
13	2.01	2.01	15.9	YEAST	Q12263
14	2	2	10.9	CHAGB	Q2GXM1
15	2	2	16.1	HUMAN	P35908
16	2	2	34	MORAP	Q9HDF5
17	2	2	11.2	ENTFA	Q833N6
18	2	2	8.6	BOVIN	P12763
19	2	2	5.1	RHOS5	A4WU98
20	2	2	38	MYCTU	P9WNK5
21	2	2	6.4	TRYBB	P12865
22	1.85	1.85	6.5	ACRMI	B3EWZ5
23	1.82	1.82	4.4	DROME	Q9VYN8
24	1.57	1.57	10.2	RABIT	P04221
25	1.39	1.39	56.7	STRM5	B4SRB3

	Protein Name	Number of Unique Peptides (C.I. 95%)
1	**5-methyltetrahydropteroyltriglutamate--homocysteine methyltransferase OS=Mycobacterium tuberculosis (strain ATCC 25618 / H37Rv) GN=metE PE=1 SV=1**	**37**
2	Ig gamma-1 chain C region, membrane-bound form OS=Mus musculus GN=Ighg1 PE=1 SV=2	42
3	Ig kappa chain C region OS=Mus musculus PE=1 SV=1	11

4	Keratin, type II cytoskeletal 1 OS=Homo sapiens GN=KRT1 PE=1 SV=6	4
5	Serum albumin OS=Bos taurus GN=ALB PE=1 SV=4	4
6	Ig kappa chain V19-17 OS=Mus musculus GN=Igk-V19-17 PE=1 SV=1	8
7	Keratin, type I cytoskeletal 9 OS=Homo sapiens GN=KRT9 PE=1 SV=3	2
8	Alpha-1-antiproteinase OS=Bos taurus GN=SERPINA1 PE=1 SV=1	2
9	Histone H2A type 2-B OS=Homo sapiens GN=HIST2H2AB PE=1 SV=3	2
10	Histone H2B 1/2 OS=Danio rerio GN=zgc:112234 PE=2 SV=3	2
11	Keratin, type I cytoskeletal 10 OS=Homo sapiens GN=KRT10 PE=1 SV=6	1
12	Antileukoproteinase OS=Mus musculus GN=Slpi PE=1 SV=1	1
13	Serine/threonine-protein kinase GIN4 OS=Saccharomyces cerevisiae (strain ATCC 204508 / S288c) GN=GIN4 PE=1 SV=1	1
14	COPII coat assembly protein SEC16 OS=Chaetomium globosum (strain ATCC 6205 / CBS 148.51 / DSM 1962 / NBRC 6347 / NRRL 1970) GN=SEC16 PE=3 SV=1	1
15	Keratin, type II cytoskeletal 2 epidermal OS=Homo sapiens GN=KRT2 PE=1 SV=2	1
16	Histone H4 OS=Mortierella alpina GN=H4.1 PE=3 SV=3	1
17	UDP-N-acetylmuramate--L-alanine ligase OS=Enterococcus faecalis (strain ATCC 700802 / V583) GN=murC PE=3 SV=1	1
18	Alpha-2-HS-glycoprotein OS=Bos taurus GN=AHSG PE=1 SV=2	1
19	Cysteine--tRNA ligase OS=Rhodobacter sphaeroides (strain ATCC 17025 / ATH 2.4.3) GN=cysS PE=3 SV=1	1
20	ESAT-6-like protein EsxB OS=Mycobacterium tuberculosis (strain ATCC 25618 / H37Rv) GN=esxB PE=1 SV=1	1
21	Bloodstream-specific protein 2 OS=Trypanosoma brucei brucei GN=BS2 PE=3 SV=1	1
22	MAM and LDL-receptor class A domain-containing protein 1 (Fragment) OS=Acropora millepora PE=1 SV=1	1
23	Teneurin-a OS=Drosophila melanogaster GN=Ten-a PE=1 SV=2	1
24	Ig mu chain C region membrane-bound form OS=Oryctolagus cuniculus PE=2 SV=2	1
25	Carbon storage regulator homolog OS=Stenotrophomonas maltophilia (strain R551-3) GN=csrA PE=3 SV=1	1

Supplementary table S3B. LC-MALDI analysis performed on the 80 kDa antigen immuno-precipitated by mAb M29

	Proteinpilot Unused Score	Proteinpilot Total Score	% of Coverage Sequence	Species	UniProtKB Accession Number
1	**44.04**	**44.04**	**42.3**	**MYCTU**	**P9WK07**
2	32	32	49.1	MOUSE	P01869
3	12.03	12.03	89.7	MOUSE	P01837
4	6	6	19.8	HUMAN	P06312
5	2	2	22.3	ORYSJ	Q8S857
6	2	2	29.8	DANRE	Q5BJA5
7	2	2	4.9	PONAB	Q5NVH5
8	2	2	8.8	GEOKA	Q5L3S9

	Protein Name	Number of Unique Peptides (C.I. 95%)
1	**5-methyltetrahydropteroyltriglutamate--homocysteine methyltransferase OS=Mycobacterium tuberculosis (strain ATCC 25618 / H37Rv) OX=83332 GN=metE PE=1 SV=1**	**25**
2	Ig gamma-1 chain C region, membrane-bound form OS=Mus musculus OX=10090 GN=Ighg1 PE=1 SV=2	31
3	Immunoglobulin kappa constant OS=Mus musculus OX=10090 GN=Igkc PE=1 SV=2	15
4	Immunoglobulin kappa variable 4-1 OS=Homo sapiens OX=9606 GN=IGKV4-1 PE=1 SV=1	5
5	Probable histone H2A variant 2 OS=Oryza sativa subsp. japonica OX=39947 GN=Os10g0418000 PE=2 SV=1	1
6	Histone H2B 1/2 OS=Danio rerio OX=7955 GN=zgc:112234 PE=2 SV=3	1
7	Serum albumin OS=Pongo abelii OX=9601 GN=ALB PE=2 SV=2	1
8	33 kDa chaperonin OS=Geobacillus kaustophilus (strain HTA426) OX=235909 GN=hslO PE=3 SV=2	1
